# ChIP analysis unravels an exceptionally wide distribution of DNA binding sites for the NtcA transcription factor in a heterocyst-forming cyanobacterium

**DOI:** 10.1186/1471-2164-15-22

**Published:** 2014-01-13

**Authors:** Silvia Picossi, Enrique Flores, Antonia Herrero

**Affiliations:** 1 Instituto de Bioquímica Vegetal y Fotosíntesis, Consejo Superior de Investigaciones Científicas and Universidad de Sevilla, Américo Vespucio 49, Seville E-41092, Spain

## Abstract

**Background:**

The CRP-family transcription factor NtcA, universally found in cyanobacteria, was initially discovered as a regulator operating N control. It responds to the N regime signaled by the internal 2-oxoglutarate levels, an indicator of the C to N balance of the cells. Canonical NtcA-activated promoters bear an NtcA-consensus binding site (GTAN_8_TAC) centered at about 41.5 nucleotides upstream from the transcription start point. In strains of the *Anabaena*/*Nostoc* genera NtcA is pivotal for the differentiation of heterocysts in response to N stress.

**Results:**

In this study, we have used chromatin immunoprecipitation followed by high-throughput sequencing to identify the whole catalog of NtcA-binding sites in cells of the filamentous, heterocyst-forming cyanobacterium *Anabaena* sp. PCC 7120 three hours after the withdrawal of combined N. NtcA has been found to bind to 2,424 DNA regions in the genome of *Anabaena*, which have been ascribed to 2,153 genes. Interestingly, only a small proportion of those genes are involved in N assimilation and metabolism, and 65% of the binding regions were located intragenically.

**Conclusions:**

The distribution of NtcA-binding sites identified here reveals the largest bacterial regulon described to date. Our results show that NtcA has a much wider role in the physiology of the cell than it has been previously thought, acting both as a global transcriptional regulator and possibly also as a factor influencing the superstructure of the chromosome (and plasmids).

## Background

Cyanobacteria are prokaryotic organisms that perform an oxygenic photosynthesis similar to that of higher plants, constituting the evolutionary ancestors of the chloroplast [1]. Cyanobacteria are generally photoautotrophs fixing CO_2_, and they preferentially use ammonium as the nitrogen source, although they can also use nitrate, urea, and some amino acids. Some filamentous cyanobacteria, such as the model strain *Anabaena* sp. PCC 7120, are also able to fix N_2_ in specialized cells called heterocysts [2]. The CRP-family transcriptional regulator NtcA, which is highly conserved in cyanobacteria, controls the response of the cell to N availability by binding to the promoter region of its target genes, activating or repressing their expression [3]. NtcA binds as a dimer to target sites with the consensus sequence GTAN_8_TAC [4]. In a number of sites found in NtcA-activated promoters binding of NtcA in vitro increases in the presence of 2-oxoglutarate (2-OG), although binding in the absence of this effector could also take place (e.g. [5]). The crystal structure of the *Anabaena* NtcA dimer has been solved in complex or not with 2-OG [6]. Similarly to CRP, each NtcA monomer comprises an N-terminal effector-binding domain and a C-terminal helix-turn-helix DNA binding domain, both connected by a long helix [6,7]. 2-OG binds at a pocket in the effector-binding domain and this binding induces changes that are transmitted to the DNA-binding domain resulting in a tighter coiled-coil conformation of the two C-helices, which is better suited for DNA binding [6]. However, whereas the apo-CRP is unable of DNA binding in the absence of cAMP, in the apo-NtcA, the conformation of the helices is permissive for DNA binding [6], consistent with in vitro DNA-binding results [7].

As the first response of the cyanobacterial cell to N starvation, NtcA activates the expression of genes involved in the scavenging of traces of combined N, such as the *nir* operon (nitrate assimilation proteins) or the *amt* genes (ammonia translocators) [8]. In filamentous, heterocyst-forming cyanobacteria, NtcA is also needed for the differentiation and function of the N_2_-fixing heterocysts in response to persistent N deprivation. The process of heterocyst differentiation is tightly regulated and involves a cascade of transcriptional regulators that is initiated by NtcA and the heterocyst-specific regulator HetR [9]. Some heterocyst-related genes activated by NtcA are *nrrA* and *hetC* (early induced), *xisA* and *devBCA* (intermediate steps), and *pipX* and the *cox2*, *cox3* and *nifHDK* operons (maturation and function) [10]. Canonical NtcA-activated promoters have a consensus NtcA-binding site, centered at about 41.5 nucleotides upstream from the transcription start point (TSP) of the regulated genes, and a -10 box with the consensus sequence TAN_3_T, thus matching the bacterial Class II activator-dependent promoters [4,11]. Genes involved in the scavenging of traces of combined N, such as *urtA*, *nirA*, *ntcB*, and *glnA*, bear canonical NtcA-dependent promoters [8]. In complex promoter regions, often found in heterocyst-differentiation genes, NtcA-dependent promoters showing a non-canonical structure have been found, such as in *cphB1* or *ntcA*[10]. Other genes such as *rbcL* or *gor*, are repressed by NtcA and have a GTAN_8_TAC box downstream of the -35 box of the promoter [4].

A prediction of new putative binding sites of NtcA in different cyanobacteria, based on computational analyses, has been published [12], but no in vivo confirmation of these sites has been done. On the other hand, two transcriptomic studies of the response of *Anabaena* sp. PCC 7120 to N deprivation have been recently published. Flaherty *et al.*[13] mapped transcripts produced in the whole genome, and Mitschke *et al.*[14] focused on possible TSPs. In order to determine the whole NtcA regulon, we attempted to find out all the NtcA targets present in the genome of *Anabaena* sp. PCC 7120 using immunoprecipitation of chromatin followed by massive sequencing (ChIP-Seq). This is a powerful technique that allows the identification of the in vivo binding sites of a transcription factor (TF) [15]. We have focused on an early time of induction after N step-down, when NtcA regulates genes involved in the scavenging of traces of combined N, but also genes required for the early stages of heterocyst differentiation.

## Results

### Immunoprecipitation of NtcA-bound DNA

Wild-type *Anabaena* sp. PCC 7120 cells growing in bubbled cultures with ammonium as the N source were subjected to incubation in a combined N-depleted medium for 3 hours, after which the cultures were treated with formaldehyde to fix the proteins bound to DNA. After cell lysis and DNA fragmentation, the extracts were treated with an anti-NtcA antibody to specifically immunoprecipitate the NtcA-bound DNA (see Methods). The immunoprecipitated material was then incubated at 65ºC to reverse the crosslinking, and the DNA was isolated. A sample of total DNA was also isolated prior to anti-NtcA treatment of the extracts to serve as the control input sample.

Quantitative PCR was performed to check the quality of the immunoprecipitated DNA, and to confirm that known NtcA target regions were enriched. Primers that amplified the promoter region of *nrrA (all4312)*, as a positive control, and the promoter region of ORF *all0770*, as a negative control, were used. The result of the Q-PCR analysis (Additional file 1: Figure S1), confirmed a substantial enrichment in the NtcA-dependent promoter. Immunoprecipitated and input DNA samples were subjected to high-throughput sequencing and the results were analyzed using the Triform algorithm [16] and mapped onto the genome of *Anabaena* sp. PCC 7120 [17] (Additional file 2: Table S1).

### Distribution of the NtcA-bound DNA throughout the genome of *Anabaena* sp. PCC 7120

The analysis of immunoprecipitated DNA showed 2,424 binding regions, all of them statistically significant, located in the *Anabaena* genome, and distributed throughout the chromosome and five of the six plasmids (Table 1; Additional file 3: Figure S2). We have analyzed the location of these binding regions on the *Anabaena* genomic sequence and assigned them to one gene, two genes (when it was not possible to decide between flanking genes), or sRNAs (Additional file 2: Table S1). The Integrative Genome Viewer program (Broad Institute) [18] was used to map the sequences of the binding regions obtained by the ChIP-Seq experiment onto the *Anabaena* genome (the data are available for its visualization at GEO accession # GSE51865). The information of the 2,424 binding regions obtained is shown in Additional file 2: Table S1, including the location in the chromosome or plasmids, the gene(s) to which the binding region has been ascribed, and the statistical significance of the peak identifying the binding region (Q value, NLQ value) (see Methods). The binding regions are about 200–250 bp long, being its midpoint the most likely position for NtcA binding. Since cyanobacterial 5′-UTRs are frequently very long [13,14], some binding regions have been assigned to genes that are relatively far, even if the binding regions were located inside the coding region of an adjacent gene. For these cases, we have taken into account the information about the differential expression of the genes after combined-N deprivation [13,14,19] (Additional file 2: Table S1).

**Table 1:** Results of the ChIP-Seq of NtcA in Anabaena sp. PCC 7120 after N step-down

**DNA**	**Size (kb)**	**Binding regions found**	**Genes ascribed**	Position of the binding region with respect to the gene*		
				**Upstream**	**Internal**	**Downstream**
**Chromosome**	6413	2143	1939	802	1548	74
**Alpha**	408	145	103	37	103	5
**Beta**	187	86	66	16	71	0
**Delta**	55.4	12	10	2	10	0
**Epsilon**	40.3	10	9	3	7	0
**Gamma**	102	28	26	5	23	0
**Zeta**	5.6	0				
**Total**	7212	2424	2153	865	1762	79

*Total number of entries in Additional file 2: Table S1 (2706) was used (some binding regions have been ascribed to more than one gene).

In order to estimate the role of NtcA binding at the binding regions, the programs CLC Sequence Viewer (CLC Bio) and Artemis Genome Browser (Wellcome Trust Sanger Institute) [20] were used to integrate the transcriptomic data from Flaherty *et al.*[13] and from Mischke *et al.*[14] with the location of the NtcA binding region sequences obtained in our ChIP-Seq experiment. To this end, the location of a certain binding region (midpoint of the range of the detected target region) (Additional file 2: Table S1) was determined using the above-mentioned programs, and its genomic context was analyzed in relation to the transcripts/transcription start sites that have been described. In addition, putative NtcA consensus binding sites were identified (if possible) within the sequence of the binding region (Table 2; Additional file 2: Table S1). The relative location of the binding region with respect to the gene was also analyzed, and the binding regions were classified into three categories: upstream from the gene (distinguishing between far upstream and close upstream), internal to the gene (close to the 5′ or the 3′ end of the gene), and downstream from the gene (Table 1 and Additional file 2: Table S1). Interestingly, about 65% of the identified binding regions were found within coding regions. Among those located in intergenic regions, most of them (865) were present upstream of the assigned genes and some (79) downstream of coding regions.

**Table 2:** Top-scoring target regions

**ID**	**NLQ**^**a**^	**Start**	**End**	**Gene**	**Description**	**NtcA bs**^**b**^	**Putative NtcA binding site and promoter sequences**^**c**^
**1203**	8186.60	3561438	3561710	alr2921*	tRNA modif. prot.	3561528	*GTGTGTCTTGATAC*AATACTTTTAAGATGACTGTTA*TATGCT*TTCTAA*G*
**1856**	7861.39	5515960	5516178	*all4613*	IlvG	5516125	*GAATTACTGACTAC*AGAATTCCCGCATTAACTTAGC*TATCAT*CTGAGGT*T*
						5516125	*GAATT* ***A*** *CTGACTAC*
**1152**	7152.53	3427339	3427611	*alr2817*	HetC [26]	3427445	***GTAACATGAGATAC*** ACAATAGCATTTATATTTGCTT ***TAGTAT*** CTCTCT ***C***
**390**	6288.90	1156154	1156424	*alr0990*	Amt4 [21]	1156258	*GTATTAACTAATAC*AGAATTAATGTTTAGGTAAATA*GACAAT*CAAT*C*
**798**	5358.67	2400656	2400888	*all2006*	Unknown protein	2400815	*GTATATTTCAACAC*GAATTTGATCATTTAGATGGTG*TACTGT*TTATAG*A*
**931**	4728.32	2807226	2807511	*alr2328*	GlnA (RNA_I_) [27]	2807342	***GTAACAAAGACTAC*** AAAACTGTCTAATGTTTAGAATC ***TACGAT*** ATTTC ***A***
					GlnA (RNA_II_) [27]	2807342	***GTAACAAAGACTAC*** *AAT*CCAGACGTTC ***T***
**1612**	4089.39	4795414	4795682	*asl3981*	Unknown protein	4795535	*G*GATTTT*GTAGCTATCACTAC*
						4795535	*GTAGCTATCACTAC*AATCTATAGCTTGAAAGAGGAG*GAAAAT*AGGTTG*G*
**293**	3663.48	815417	815662	*alr0709*	Fe-responsive RR [25]	815545	*GTATAAATTTTTAC*
**364**	3580.62	1070920	1071178	*all0926*^*§*^	PilL	1071039	*GTAGCCCCTGCTAC*
**1764**	3570.57	5243203	5243472	*all4376*	SpsB [28]	5243396	*GTATTGAAAATTAC*AAAAATCTTATTTACTTATTAG*TAACAT*TGGTGA*C*
**1165**	3408.52	3463447	3463731	*all2842*^*§*^	Unknown protein	3463590	*GTTACACCTGCTAC*
**1125**	3369.20	3341106	3341375	*alr2743*	Processing protease	3341205	*GTATAACTTGATGC*GAATTTGGCGATCGTTCAGAGT*TAAGAT*CACAAAC*A*
**1530**	3353.43	4573161	4573410	*asl3784*	hypothetical protein	4573340	*GTTAAATAGATTAC*AAAACTCTGGATGGACTTTGAG*TATCAT*TTAGAT*A*
**932**	3292.44	2809205	2809466	*asl2329*	GifA [29]	2809350	***GTAGCATAAGATAC*** AGAATTCTTGC ***TATATT*** AAATGT ***G***
**2098**	3220.20	6294152	6294404	*alr5275*	Gnd	6294223	*GTTGATTTGGATAC*AAATTAAAACTATTTATCTGTGTTACTAGTGAGCTTTT*A*
				*all5274*	Hypothetical protein	6294223	*GTATCCAAATCAAC*AATTAACTTG*TAAAAT*TTGCG*A*
**319**	3192.18	902825	903070	*all0778*	Ser protease inhibitor	902941	*GTAGCCATGAATAC*
**1150**	3126.82	3420486	3420738	*alr2811*	AvtA	3420576	*GCTGAGATTGGTAC*
**2232**	2974.32	264949^†^	265189^†^	*alr7243*^*§*^	Similar to ankyrin	265067^†^	*GTAGCCTGTGATAC*
**898**	2824.47	2729431	2729698	*all2267*	Hypothetical protein	2729550	*GTATTGCTGGCTAC*
**1858**	2807.34	5523157	5523405	*all4620*^*a*^	Na^+^/H^+^-exch. prot.	5523288	*GTATAAGCTGTAAC*
**1255**	2798.00	3732951	3733219	*all3084*	Hypothetical protein	3733071	*GTTAAAATGACGAC*ATTTTAATAAAAAGAAGCA*TATACT*CGATGT*A*
**260**	2698.90	701137	701391	*all0602*	NtcB [30]	701297	***GTAACAAAATCTAC*** CAAATTGGGGAGCAAAATCAGC*TAACTT*AATTGA ***A***
**377**	2641.34	1111093	1111337	*all0956*	Unknown protein	1111206	*GTAGCATAGACAAC*
				*alr0957*	FurC [31]	1111206	*GTAGCATAGACAAC*
**519**	2592.23	1576957	1577227	*asr1328*	Hypothetical protein	1577115	*GTAACATACACTAC*GAAACTTATGC*TATGTT*AGGAAGA*A*
**2149**	2572.01	23295^†^	23563^†^	*all7026*^*§*^	DNA binding protein	23435^†^	*GTATTCGCTGATAC*
**554**	2517.34	1669629	1669873	*alr1404*	Ser acetyltransferase	1669702	*GTGACTGGGGATAC*AGAAAATTTATTTATAGTCCTGTC*TACTGT*ATATT*C*
**1784**	2362.05	5312632	5312908	*all4432*^*§*^	EPS biosynthesis	5312767	*GTATCAACTGCTAC*
**1660**	2280.02	4946500	4946770	*alr4105*^*§*^	2-comp. sensor HK	4946621	*GTAGCAGGTTATAC*
**2345**	2253.01	105303^‡^	105553^‡^	*all7614*	Putative porin, OprB-II	81139^‡^	*GTATGAAATAGTAC*AGTTTAAAATTAGTGTTTGCGT*CATCAT*TACGAG*A*
**38**	2141.66	69752	69991	*asr0064*	Hypothetical protein	69882	*A*GTTCC*GTATAGCAAAATAC*
**1118**	2134.47	3325955	3326229	alr2729*	*cox3* operon	3326057	*GTTACTAAAGATAC*AGTATTTTCTAGCTTTTAAATT*TTGAAT*ACAAAG*A*
**1283**	2115.10	3807024	3807271	*all3144*	Hypothetical protein	3807189	*GTATCGACCATTAC*
**94**	2063.99	210284	210541	*alr0194*	Hypothetical protein	210417	*GTATTACCTAATAC*
				*all0193*	Haloalkane dehalogenase	210417	*GTATTACCTAATAC*
**1316**	1948.08	3903915	3904189	*all3232*	2-comp. RR	3904009	*GTAACCAAATATAC*
				*alr3233*	TrpE	3904009	*GTAACCAAATATAC*
**262**	1927.75	704040	704304	*alr0607*	NirA [32]	704169	***GTAGCTACTTATAC*** TATTTTACCTGAGATCCCGACA ***TAACCT*** TAGA ***A***
**729**	1908.73	2158958	2159198	*all1797*	Unknown protein	2159129	*GTATCCTCTGCTGC*TGCACTAGAAGTTTGTTCAAGG*TTTAAT*TGTAAT*A*
**1923**	1885.93	5718871	5719130	*alr4800*	Anti-σ antagonist	5718952	*GATACGATTGATAC*CGAAATCCTTAGGCGTTTGTGCC*TATATT*GAGAAA*A*
**2111**	1840.96	6328022	6328296	*alr5307*	Glycosyl transferase	6328115	*GTAGTTTTCGCTAC*
				*all5306*	Exodeoxyribonuclease III	6328115	*GTAGTTTTCGCTAC*
**695**	1791.79	2058723	2059007	alr1713*	Hypothetical protein	2058829	*GTAACCTATAAGAC*ATTTTATTTGATACCTCATACTC*TAAAAT*CAAGT*A*
**776**	1744.65	2304064	2304320	*alr1921*^*§*^	Biotin synthase	2304193	*GAAGTGTGCTGTAC*

†Genomic positions within the alpha plasmid. ‡Genomic positions within the beta plasmid.

^§^Target regions located in internal positions of the ascribed genes. All the other target regions are upstream of the ascribed genes. The genes marked with * could be ascribed to sRNAs located in the upstream region of the indicated genes.

^a^NLQ: Indication of the statistical significance of the peak identifying the binding region.

^b^Position in the chromosome/plasmids of the first nucleotide of the putative NtcA binding sites.

^c^The putative NtcA binding sites, -10 boxes and TSPs are in italics. Those that have been previously described are indicated in bold. In some cases, the detected NtcA-binding site could be associated to two TSPs, either of the same gene or of two divergent genes.

### Functional categories of genes with NtcA-binding sites

The genes identified as NtcA targets 3 h after N step-down have been classified into eight functional categories (Table 3). About half of the assigned genes (1,074) encode proteins with unknown function. Within the genes encoding proteins involved in cellular processes (234 genes), 90 were translation-related genes, including several ribosomal proteins, and 22 were related to transcription. Among the genes encoding proteins involved in N metabolism and N fixation, 33 genes were involved in N scavenging and metabolism, including well-known NtcA-dependent genes such as *ntcB*, *nirA*, *amt4*, *cphB*, etc. [4,8,21].

**Table 3:** Functional categories of the assigned genes

Category*	**Number of genes**
**Cellular processes**	**234**
Translation	90
Transcription	22
DNA replication, recombination, and repair	39
Cell killing	5
Cell envelope	44
Others	34
**Metabolism**	**151**
Purines, pyrimidines, nucleosides, and nucleotides	25
Fatty acid, phospholipid and sterol metabolism	16
Central metabolism	55
Biosynthesis of cofactors, prosthetic groups, and carriers	55
**Nitrogen metabolism and nitrogen fixation-related**	**121**
Amino acid biosynthesis	44
Nitrogen metabolism	33
Heterocyst differentiation and function	44
**Regulatory functions**	**179**
Transcriptional regulators	28
Serine/threonine kinases	18
Two-component hybrid sensor and regulators	29
Two-component response regulators	22
WD-proteins	15
Two-component sensor histidine kinases	32
Serine/threonine kinases with two-component sensor domains	11
Phosphatases	3
Others	21
**Photosynthesis and respiration**	**57**
**Transport and binding proteins**	**124**
Iron-related genes	30
**Other categories**	**213**
Hydrogenases	7
**Hypothetical proteins**	**1074**

*The eight main categories in which the assigned genes have been classified are indicated in bold.

A total of 179 genes encoding regulatory proteins were found, some of which were already known NtcA-targets, including genes encoding the iron-responsive transcriptional repressor FurA [22], the regulatory factor PipX [23], the two-component response regulator NrrA [24], and the *pkn41* and *pkn42* genes encoding Ser/Thr kinases with two-component sensor domains [25]. Some of the newly identified regulatory genes with NtcA binding regions were those encoding the transcriptional repressor SmtB (present in the beta plasmid), the two-component response regulator RpaA, or the Ser/Thr phosphatase Alr3732. Interestingly, the proportion of internal NtcA binding regions within the genes encoding regulatory proteins was higher than in other categories of genes. Regarding metabolism-related genes, there were 55 from the central metabolism, including carbon-assimilation genes, and genes activated (e.g. *zwf*), or repressed (e.g. *rbcL*), in the heterocysts [10].

### Analysis of binding regions with highest NLQ value

All the binding regions identified by ChIP-Seq analysis had extremely low Q values due to the exceptional quality of the deep sequencing of the immunoprecipitated DNA. We have used the NLQ value to analyze the binding regions, which allowed us to sort those regions with Q value close to zero. A list of the 40 most represented binding regions (corresponding to highest NLQ values) is shown in Table 2. Within these target regions, we localized putative NtcA-binding sequences, which in most cases were found around the midpoint of the target region and, whenever possible, they were correlated with previously described TSPs (all of which were differentially expressed in N-depleted conditions) by comparison with the data from Flaherty *et al.*[13] and Mitschke *et al.*[14], as well as from individual analyses [21,25-32].

**Figure 1:**
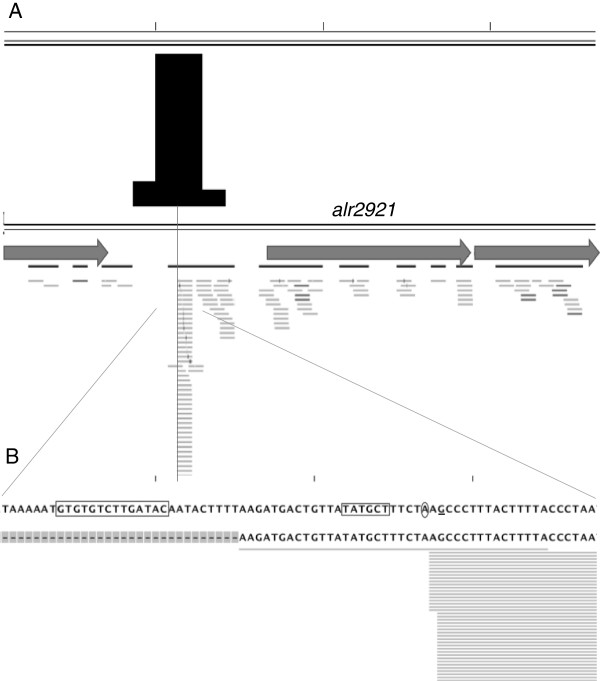
**Nucleotide analysis of the top-scoring target region in the ChIP-Seq experiment showing the presence of an NtcA-binding site. A)** Location of the target region (in black) with respect to the adjacent ORFs and in the context of the transcriptomic data from Flaherty *et al.*[13]. **B)** Zoomed image of the midpoint of the target region (circled A), the putative TSP (G), and the putative NtcA-binding sequence (GTGTGTCTTGATAC) located 22 nucleotides from a putative -10 box (TATGCT) (CLC Sequence Viewer). Horizontal gray lines indicate transcription [13].

Some target regions were included in DNA sequences that conform Class-II NtcA-dependent promoters. Among these is the target region with highest NLQ value (#1203), which was located upstream of the ORF *alr2921*, with a GTGN_8_TAC sequence separated 35 nucleotides from a bulk of 5′-transcript ends activated in combined-N free medium [13], which could represent a putative TSP, preceded by a -10 box (Figure 1). In others, the target region was included in DNA sequences showing a repressor-like position. Finally, in other target regions only a putative binding site could be found with no correlation with any known TSP. In some cases one binding site could be affecting the expression of two TSPs (both of the same gene or of two divergent ones), and in others the putative binding site could be ascribed to two genes (with no apparent relation with any TSP). The target region with second highest NLQ value (region #1856) was located upstream of the *ilvG* gene encoding an enzyme of the biosynthesis of the branched chain amino acids, which is slightly repressed in combined N-free medium [13,19]. The NtcA binding site identified in this target region, GAAN_8_TAC, overlaps a TSP that is repressed under N depleted conditions (Table 2 and [14]). In addition to the repressor effect described above, the NtcA binding sequence upstream of *ilvG* is separated by 22 nucleotides from the -10 box of an N-dependent activated TSP, thus representing a putative Class II promoter activator site. This is similar to what we have found for the target region located upstream of *glnA*, which has already been described (Table 2 and [27]).

**Figure 2:**
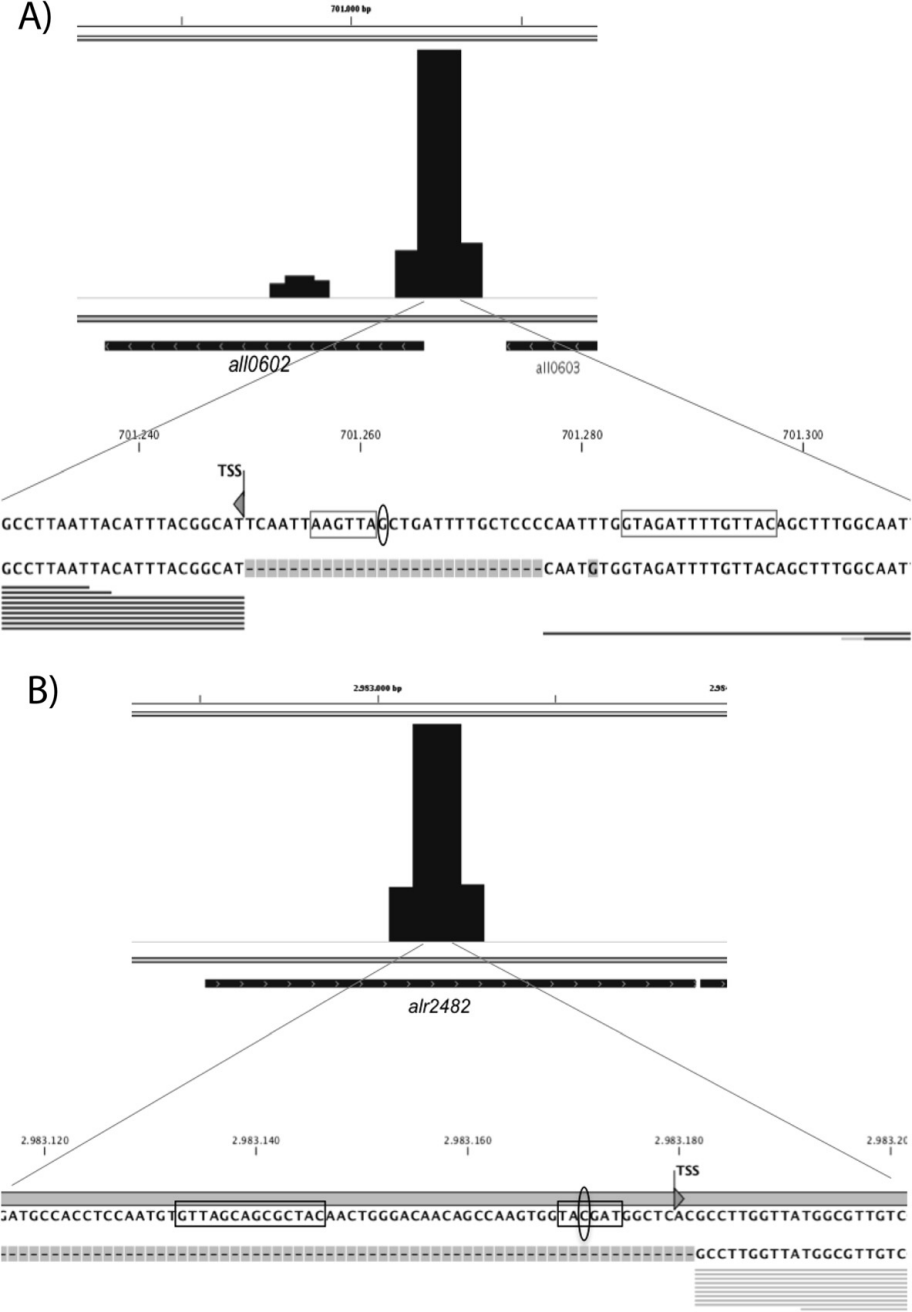
**Nucleotide analysis of two high-score target regions. A)** Promoter sequence of *ntcB* (*all0602*), including the NtcA binding site, the -10 box and the TSP (or TSS) (already described) [30], and the midpoint of the target region found in the ChIP-Seq experiment (circled G). **B)** Analysis of the sequence of the target region found inside ORF *alr2482* (midpoint of the target region represented by the circled C), which includes a putative NtcA binding site separated 22 nucleotides from a putative -10 box of an internal regulated TSP [14]. Transcriptomic data was obtained from [13] (12 h after N withdrawal).

Figure 2 shows two examples of target regions whose sequences contained a Class-II NtcA-dependent promoter structure. One is the well-known NtcA-dependent gene *ntcB*, whose promoter determinants have already been described [30] (Figure 2A and Table 2). The other example is a high-NLQ target region mapped inside the ORF *alr2482* (target region #1165), whose sequence also showed a Class-II NtcA-activated promoter structure that could be regulating an internal TSP activated under N deprivation [14].

### In vitro binding of NtcA

In order to corroborate our results, we first checked whether our ChIP-Seq analysis identified already known NtcA binding sites located in promoter regions of genes regulated by this transcription factor. Indeed, Additional file 4: Table S2 shows eighteen target regions indentified in this work which include NtcA binding sites identified previously by different means, such as those corresponding to the nitrate assimilation genes *nirA* and *ntcB*, the ammonium assimilation gene *glnA*, and the heterocyst-related genes *hetC*, *xisA* and *devB*, among others.

**Figure 3:**
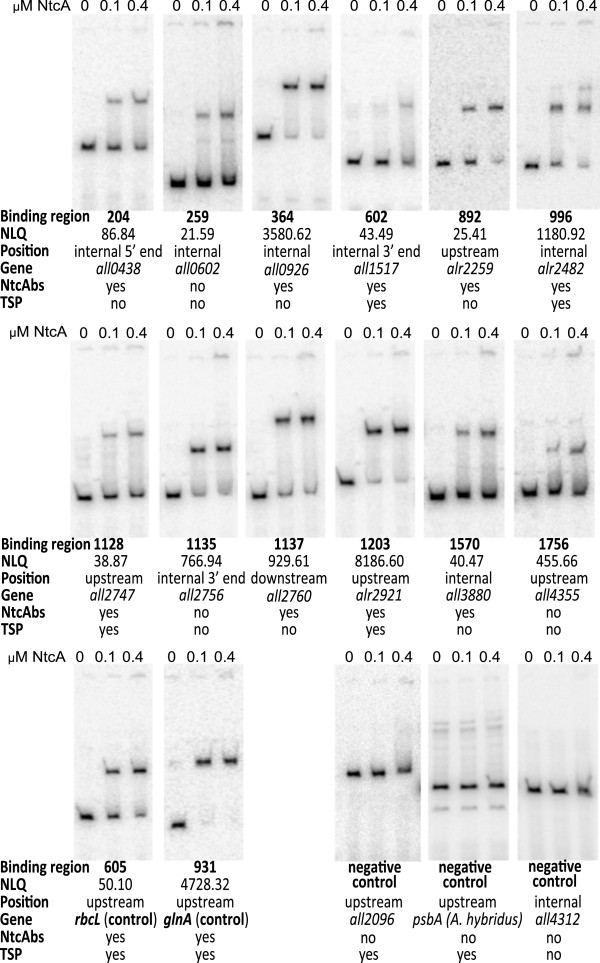
**Electrophoretic mobility shift assay of several binding regions.** Results of the EMSA carried out using DNA fragments from the indicated binding regions tested, using the indicated NtcA concentrations. The NLQ value, position with respect to the gene, the ascribed gene, the NtcA binding site identified, and the presence of a TSP are indicated.

Additionally, electrophoretic mobility shift assays (EMSA) were performed to check the capability of NtcA to bind in vitro to DNA fragments included in different binding regions (Figure 3) identified in this study. Target regions #605 and #931 (which include the NtcA binding sites located upstream of *rbcL* and *glnA*, respectively [Additional file 2: Table S1]) were used as positive controls. As negative controls, we used a DNA fragment upstream of *all2096*, an internal region of *nrrA*, and the promoter region of the *Amaranthus hybridus psbA* gene, which have not been described as NtcA targets. A selection of twelve binding regions with different features were tested. We included binding regions with low NLQ (#204, 259, 602, 1128 and 1570), and with high NLQ (#364, 996, 1135, 1137, 1203 and 1756). The analyzed binding regions were located intragenically (#204, 259, 364, 602, 1135 and 1570), upstream (#892, 996, 1128, 1203 and 1756), or downstream (#1137) of genes. A putative consensus NtcA binding site was identified in binding regions #204, 364, 602, 892, 996, 1128, 1137, 1203 and 1570, while binding regions #602, 996, 1128 and 1203 were found close to a TSP (Class II-dependent activation compatible sites) (Figure 3). Several concentrations of NtcA were used in the EMSA assays and, in general, there was a correlation between NLQ and the affinity of NtcA for the binding region, which indicated the quality of the results. NtcA showed a higher affinity for binding regions with high NLQ than for those with low NLQ, regardless of the position of the binding regions with respect to the genes.

### Consensus NtcA-binding site

**Figure 4:**
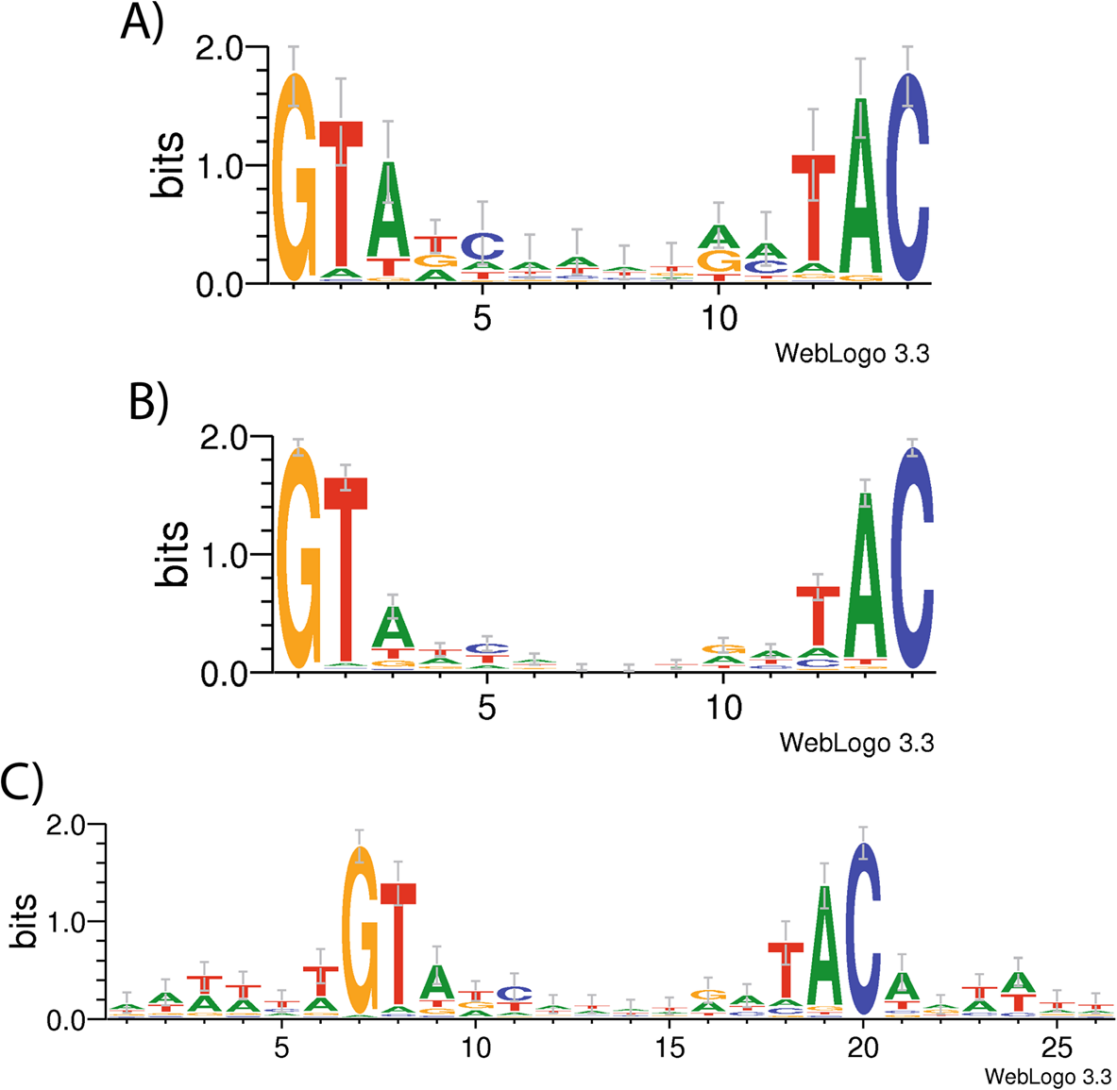
**Consensus NtcA-binding site. A)** Consensus NtcA-sequence derived from the 40 target regions with highest NLQ (Table 2). **B)** Consensus binding site found using all the putative NtcA binding sequences in Additional file 2: Table S1 (508 sequences). **C)** Extended consensus NtcA binding site (135 sequences). (Representations by WebLogo, Berkeley) [33].

An initial CisFinder analysis of the whole data resulting from the deep-sequencing of the immunoprecipitated DNA, using randomly located subsequences as control, showed a predominant target region-specific motif: GTAN_8_TAC. We have done additional analyses of the NtcA binding sequences identified using the Weblogo application [33] (Figure 4). The consensus sequence of three different groups of NtcA binding sites have been analyzed. The first included the putative NtcA sequences identified in the 40 target regions with highest NLQ (Figure 4A). The well-known NtcA-binding site sequence [4] was obtained, having somewhat conserved T and C nucleotides in positions 4 and 5, and A in positions 10 and 11. When the 508 putative NtcA sequences identified (mainly located in the target regions with highest NLQ values; Additional file 2: Table S1) were analyzed, the NtcA consensus binding site shown in Figure 4B was obtained. In this case, the GTN_10_AC was highly conserved, with also some prevalence of an A at position 3 and a T at position 12. Finally, we did an analysis of the extended NtcA binding site (i.e. including 6 nucleotides upstream and 6 nucleotides downstream of the GTAN_8_TAC core). In this case, a total of 135 sequences were used, obtained either from the target regions with highest NLQ or those NtcA binding sites that were associated with a promoter (indicated in Additional file 2: Table S1). A clear conservation along a 24 nt-long sequence was observed, specially the occurrence of A/T pairs in positions 3, 4, 6, 21, 23 and 24 (Figure 4C), consistent with previous observations [4,11].

## Discussion

NtcA is a transcriptional regulator of the CRP family that has been described to act upstream of the regulated genes, usually as an activator, but also as a repressor [3]. By ChIP-Seq analysis we have found that NtcA binds to up to 2,424 genomic sites, including sites in the chromosome and five of the six plasmids present in *Anabaena* sp. PCC 7120. The fact that no binding regions were found associated to plasmid zeta is probably due to the small size of this plasmid (only 5.6 kb and 5 genes). The 2,424 NtcA targets found by ChIP-Seq analysis have been ascribed to 2,153 genes. A numerous group of already-known NtcA-binding sites have been identified in the target regions found in this study (see Additional file 4: Table S2), which validates the results of the ChIP-Seq analysis. Additionally, we have found that NtcA binds in vitro to a selection of target regions with different features (different NLQ, different positions with respect to the genes, etc.) (Figure 3), which further indicates that the target regions identified by ChIP-Seq are bona-fide targets of NtcA. It is worth noting that point mutations in DNA sites matching the consensual sequence GTAN_8_TAC affect NtcA binding, as it has been previously shown for the promoter region of *glnA* (corresponding to target region #931) [27] and the two NtcA binding sites of the *ntcA* promoter (corresponding to target region #1776) [5].

A very high proportion of target regions have been found located in internal positions of the genes and even downstream of coding regions (Table 1 and Additional file 2: Table S1). Not only our results with NtcA in *Anabaena* sp. PCC 7120, but many other examples show that regulation by transcription factors is much more complex than the canonical model. One fourth of the binding sites of *S. enterica* OmpR [34] and 42% of the binding sites for *C. glutamicum* GlxR [35] were found in intragenic regions. By binding to intragenic target regions, NtcA could act canonically by regulating internal TSPs as a Class II activator. In fact, some internal target regions found in our ChIP-Seq experiment seem to include sites regulating internal TSPs, both in the same orientation as the genes or counter-oriented (according to refs. [13,14]), which may correspond to overlapping as yet non-annotated genes, to antisense transcripts, or to non-coding transcription. Some examples of internal target regions in which NtcA would be acting canonically are #1431 (*alr3522*), #2367 (*alr7649*), #441 (*alr1155*), or #996 (*alr2482*) (see Table 2; Figures 2 and 3; Additional file 2: Table S1). Another possibility for the intragenic target regions is that NtcA could act as a repressor by blocking transcription elongation, as it has been shown for CodY in *B. subtilis*[36]. NtcA could be also regulating the promoters of adjacent genes with long 5′-UTRs. Finally, NtcA could be acting non-canonically by binding to intragenic regions in which not even a consensus NtcA binding site can be found, such as target regions #1135 (*all2756*) or #259 (*all0602*), for which we have detected binding of NtcA in vitro (Figure 3). In this context, it is worth noting that NtcA has been shown to regulate the proximal promoter of *devB* by binding to a site (GTCATCTAAGTTGC) quite deviated from the consensual NtcA binding sequence [37], and so it is possible that, in some cases, NtcA acts as a transcriptional regulator by binding to sequences that we are not able to predict as NtcA-binding sites.

By means of ChIP-chip analysis in *E. coli*, the global transcriptional regulator CRP has been shown to bind to 68 high-affinity sites, while showing an extensive background binding that the authors interpret as low-affinity interaction of CRP to thousands of sites [38]. The authors suggest that CRP acts, in addition to directly regulating transcription, as a chromosome-shaping protein by binding to those multiple low affinity sites. On the other hand, ChIP-Seq analyses carried out with 50 TFs of *Mycobacterium tuberculosis* in which binding sites are found in diverse genomic locations suggest that modulation of DNA structure might be a general role of TFs [15]. Our data suggests that NtcA could act similarly in those sites where it binds with a low affinity and for which no consensus-binding site can be found. There are 752 binding regions with NLQ<30, 551 of which are located in internal or downstream positions (76%), and only 179 (24%) upstream of genes. The role of NtcA as a chromosome-shaping factor instead of CRP would be consistent with a more restricted role of CRP-like proteins in the obligate photoautotroph *Anabaena* sp. PCC 7120 [39].

The high number of target regions not directly associated to TSPs could be reflecting an additional level of complexity, such as of long-range interactions of NtcA with the regulated promoters [15]. This would especially apply to internal target regions, to those located upstream but far from the coding region, and to regions located downstream of genes. Long-range effects of a TF can be achieved by cooperative binding to several binding sites or by DNA looping. In this case, the low-affinity sites would be acting cooperatively together with other, high-affinity, sites [15]. There are some individual examples of regulation at atypical distances, such as that carried out by *B. subtilis* RocR, which regulates *rocG* expression by binding to an enhancer located 1.5 kb downstream of the *rocG* promoter [40], or that by *E. coli* NtrC, whose binding sites at the σ^54^-dependent promoter of *glnA* can be located up to 2 kb away [41]. Some putative examples of long-range effects of NtcA could be two high-NLQ target regions in which a consensus NtcA-binding site can be identified, target regions #364 (*all0926*) and #1137 (*all2760*) (see in vitro binding in Figure 3), but no obvious relation with a promoter can be found.

It is interesting to note the prediction that the higher the NLQ the more NtcA protein binding to a particular sequence. Indeed, we have seen a correlation between the NLQ and the affinity of NtcA for the target region in vitro (Figure 3). According to Galagan *et al.*[15], differences in the probability of occupancy of a TF are influenced by several factors including the concentration and modification state of the TF, the affinity of the TF for the binding site, the accessibility of the binding site, and the availability of molecular co-factors. In organisms with different cell-types, more complexity can be added, since there are differences in gene regulation between different cell types. This is the case of *Anabaena* sp. PCC 7120 growing in a combined N-free medium, whose filaments contain vegetative cells and differentiating and mature heterocysts.

The 2,153 genes associated to the 2,424 binding regions found in this ChIP analysis have been classified according to the functional category of their protein products. These include known targets of NtcA involved in the scavenging of traces of combined N, such as the *amt* genes, *ntcB*, and the *nir* and *urt* operons, as well as other targets, such as the *cphBA1* operon, *hetC* and *nrrA*, which are also activated early upon N step-down (Additional file 2: Table S1). Interestingly, however, only a small fraction of all the ascribed genes are involved in N metabolism (including N scavenging and amino acid metabolism) and in differentiation and function of heterocysts (5.6%). In contrast, a similar or even higher proportion of genes related to regulatory (8.2%) and transport (5.8%) functions (excluding genes related to N metabolism and transport) were found. It is also noticeable that there is a substantial number of genes involved in translation, including several ribosomal protein-encoding genes (4.2%), genes of the central metabolism of the cell (2.5%), and genes involved in the biosynthesis of cofactors, prosthetic groups and electron carriers (2.6%). Finally, ca. 50% of the ascribed genes in the ChIP-Seq analysis correspond to hypothetical or unknown proteins (Table 3; Additional file 2: Table S1). Interestingly, besides the already known NtcA targets *furA*, *pkn41* and *pkn42*[22,25], which are involved in the regulation of iron acquisition-related genes, there are about 30 iron acquisition-related genes that appear to be directly regulated by NtcA (Table 3). These results represent an in vivo corroboration of a cross-talk regulation between N and Fe metabolisms that has been proposed to happen in *Anabaena* sp. PCC 7120 [31]. On the other hand, a substantial group of genes involved in carbon metabolism are also found ascribed to target regions. These include the pentose-phosphate pathway genes *gnd*, *alr0782*, *alr4670* and *all2563*, the Calvin cycle *rbcLXS* operon and *all4021*, and many others (see Additional file 2: Table S1), such as the *cmpR* gene, whose product activates the expression of the *cmp* bicarbonate transporter genes and is regulated by NtcA [42]. These results support the idea of NtcA as a regulator of carbon assimilation genes, as previously suggested [12,42]. Finally, there is a numerous group of photosynthesis- and respiration-related genes associated to target regions, such as several genes encoding NADH dehydrogenases and ATP synthases, genes of the photosystem I and photosystem II, and several terminal respiratory oxidase-encoding genes, including the previously described NtcA-dependent *cox2* and *cox3* operons [8] (see Additional file 2: Table S1). It is interesting to note that there are twice as many ascribed N metabolism-related genes among the target regions with Q value=0 than within all the target regions (11.9% vs. 5.6%) (see Table 3; Additional file 2: Table S1; Additional file 5: Table S3). In addition, 75% of the high-NLQ target regions ascribed to N-metabolism related genes were located in positions upstream of coding regions. This is consistent with the fact that NtcA was originally discovered in studies of regulation of N assimilation [4].

Many genes that were expected to be activated later on after N step-down have been detected to be bound by NtcA in vivo at 3 h after combined-N deprivation. Some are involved in heterocyst differentiation and function, such as *xisA* or *devBCA* (intermediate steps), but also *pipX* and the *cox3* and *nifHDK* operons (late stages), which are already known targets, as well as others such as some genes of the *hep* island, *hgdD*, *nifV2*, *nifS*, *hepB*, *hepK*, *hetM*, *hetN*, and genes encoding several heterocyst-glycolipid synthases (*hglC*, *hglD*, *hglG*) (Additional file 2: Table S1). These results indicate that NtcA binds in vivo to genes that have not been shown to be differentially expressed as soon as 3 h after N step-down. In some cases, including those target regions with NLQ<100, NtcA could bind with low affinity, so that relevant binding would require a higher concentration of NtcA, an additional factor, or both (see Figure 3). The *ntcA* gene is maximally expressed at ca. 9 h upon N step-down [10]. It is possible therefore that binding of NtcA can be detected as early as 3 h upon N step-down, but this binding would not be sufficient to activate the expression of those genes until the concentration of NtcA increases. It can also be the case, especially for those targets with high NLQ, that the process of gene activation/repression starts earlier than it can be detected by conventional procedures.

A computational analysis of cis-regulatory elements located upstream of coding regions carried out by Su *et al.*[12] predicted 106 putative NtcA binding sites in the genome of *Anabaena* sp. PCC 7120. A comparison of these putative sites with our in vivo results rendered 39 common sites (Additional file 6: Table S4), including three in photosynthesis-related genes (*alr0021*, *all3410* and *all2327*) and several in N-scavenging genes (*nirA*, *ntcB*, *amtB*, and *urtA*). On the other hand, a sequence analysis of upstream regions of highly induced or repressed TSPs carried out by Mitschke *et al.*[14] predicted 115 putative NtcA binding sites. This analysis was based on a search of the consensus binding site of NtcA in regions near TSPs that were highly regulated at 8 h after N-withdrawal. Additional file 6: Table S4 shows a comparison between these sites and the NtcA binding sites identified by our in vivo ChIP-Seq analysis, which rendered 53 sites in common, including some located within coding regions.

The high number of binding regions found as well as the broad distribution of the ascribed genes in functional categories indicate that NtcA has a much wider role in the cyanobacterial cell than it had previously been assigned. Moreover, the fact that a high number of genes involved in regulatory functions appear to be regulated by NtcA implies an even broader role of this protein, since further genes may be indirectly regulated by NtcA. In spite of the binding regions with NLQ<30 (752) at which NtcA might be acting as a chromosome shaper, NtcA is the bacterial transcription factor for which the largest direct regulon has been identified by in vivo ChIP experiments. *Corynebacterium glutamicum* GlxR, a cAMP-dependent CRP-type global transcriptional regulator, has been described to bind up to 239 sites [35], while others with a less wide role, such as *Mycobacterium tuberculosis* LexA or *Salmonella enterica* OmpR, have been found to bind 25 and 58 sites, respectively [34,43].

## Conclusions

The NtcA regulon identified here constitutes the largest bacterial regulon described to date. Although initially identified in studies of regulation of N assimilation, we have shown by in vivo ChIP-Seq that NtcA has a much wider role in the physiology of the cell. NtcA can have a genomic-wide effect both as a possible chromosome (and plasmid) shaper and as a global transcription factor in the cyanobacterial cell.

## Methods

### Culture induction and formaldehyde treatment

Cells of *Anabaena* sp. (also known as *Nostoc* sp.) strain PCC 7120 growing exponentially (3-5 μg Chl/ml) in the light (75 μE·m^-2^·s^-1^) at 30°C in BG11_0_ medium [44] supplemented with 10 mM NaHCO_3_ (referred to as BG11_0_C) containing 6 mM NH_4_Cl and 12 mM TES and bubbled with a mixture of air + 1% CO_2_ were collected, washed with BG11_0_C, resuspended in BG11_0_C, and incubated in the same conditions for 3 h. Formaldehyde was then added to the cultures to a final concentration of 1%, and the cultures were incubated for 15 min (no aeration, occasional shaking). Glycine was added at 125 mM final concentration and the incubation was continued for 5 min to stop the fixing reaction. The cells were then filtered, washed with cold TBS (20 mM Tris–HCl, pH 7.4, 140 mM NaCl) and collected in tubes (25 ml of culture per tube). The pellets were frozen in liquid nitrogen and stored at -20°C until used.

### Cell lysis and DNA shearing

Cells corresponding to about 150 ml of culture (6 tubes) were used for each ChIP experiment. Pellets corresponding to about 25 ml of culture were resuspended in 500 μl of lysis buffer (50 mM HEPES/KOH, pH 7.5, 140 mM NaCl, 1 mM EDTA, 1% Triton X-100, 0.1% sodium deoxycholate, supplemented with Mini EDTA-free protease inhibitor cocktail [Roche]). Cells were supplemented with 150 μl of glass beads (acid-washed, 425-600 μm [Sigma]) and broken in a multivortexer at 2000 rpm for 1 h at 4°C. The cell lysates were collected by centrifugation and the extracts were subjected to sonication to shear the DNA into about 200-bp fragments (40 cycles of 10s, 30s ice, 10% amplitude, in a Branson Digital Sonifier). After centrifugation to eliminate cell debris, the whole-cell extracts were stored at -20°C or immediately used for immunoprecipitation.

### Chromatin immunoprecipitation

Immunoprecipitation of DNA was carried out as described in Hanaoka and Tanaka [45], with some modifications [46]. Whole-cell extracts were prepared at 4 mg/ml of total protein with lysis buffer (in 500 μl total volume). A 50-μl sample was taken as the input sample, and the extracts were pre-treated with 0.6 mg of lysis-buffer-equilibrated Dynabeads Protein G (Invitrogen) (to avoid non-specific binding of DNA to the Protein G). Anti-NtcA antibody (or H_2_O for the mock sample) was added and incubated at 4°C with rotation overnight. The extracts were treated with 0.6 mg of Dynabeads Protein G for 2 h at 4°C with rotation. The Dynabeads were washed twice with 1.5 ml of lysis buffer (5 min, rotation), and once with 1.5 ml each buffer 1 (lysis buffer containing 500 mM NaCl), buffer 2 (10 mM Tris–HCl, pH 8, 250 mM LiCl, 0.5% NP-40, 1 mM EDTA), and buffer 3 (10 mM Tris–HCl, pH 7.5, 1 mM EDTA, 50 mM NaCl). The Dynabeads were resuspended in a solution of DNase-free RNase A (0.2 μg/μl in TE), incubated for 30 min at 37°C, and washed with 1.5 ml wash buffer 3. To elute the immunoprecipitated material, the Dynabeads were resuspended in 50 μl of elution buffer (50 mM Tris–HCl, pH 7.5, 10 mM EDTA, 1% SDS) and incubated at 65°C for 30 min. The elution step was repeated once and the two eluates were combined.

### Crosslinking reversion and DNA isolation

For crosslinking reversion, the eluted material was incubated at 65°C for 5 h. The input sample was processed in parallel (Tris–HCl, pH 7.5, EDTA and SDS was added to reach the same concentration as in the ChIP sample). To eliminate proteins, Proteinase K was added at 0.4 μg/μl (final concentration) and the mixture was incubated for 1 h at 55°C. DNA was purified by phenol/chloroform/isoamyl alcohol extraction (25:24:1) followed by two extractions with chloroform/isoamyl alcohol (24:1). DNA was ethanol-precipitated using ammonium acetate and glycogen, and the pellet was washed twice with 70% ethanol, air-dried and resuspended in 25 μl purified H_2_O. For ChIP-Seq DNA samples, this protocol was repeated three times using cells from independent inductions, and the resulting DNA was mixed together and concentrated to 25 μl.

### Massive sequencing of the immunoprecipitated DNA

Input and ChIP DNA samples were sent for sequencing at the Functional Genomics Core Facility of the Institute for Research in Biomedicine, Barcelona (Spain) (Herbert Auer). Next generation sequencing was carried out using Illumina’s sequencing technology. ChIP DNA Sample Prep Kit (Illumina) was used for library preparation. Libraries were loaded at 8 pM concentration into the flow cell using the Cluster Station running recipe V7 with the Single-Read Cluster Generation Kit v4 (all Illumina). The flow cell was loaded into the Genome Analyzer II and samples were sequenced for 120 nucleotides from a single end using the Sequencing Kit v5 and recipe v8 (all Illumina). Manufacturer’s recommendations were strictly followed. Illumina sequencing data were pre-processed with the standard Illumina pipeline version 1.5 and sequences were aligned to the *Anabaena* sp. PCC 7120 genome (http://genome.microbedb.jp/cyanobase/Anabaena) with the Bowtie software 0.12.5 [47]. The percentage of reads mapped to the genome was 92.3% for the Input sample (HQ reads: 30,192,934, 64.9% of total) and 94.2% for the ChIP sample (HQ reads: 31,352,138, 68.5% of total).

The analysis of the results was carried out using the Triform algorithm method [16] (Karl Kornacker). For detected double-strand peak regions, the peak locations were reported as the averages of the forward and reverse peak locations; the z-scores were calculated according to equations (4) - (6) [16], with C (x) being replaced by the sum of the coverages on the forward and reverse peak locations; and the associated discrete p-values were adjusted for multiple testing by application of the Tarone-modified distribution-free Benjamini-Yekutieli method, similar to a method recommended in Gilbert, 2005 [48]. The Q value measures the statistical significance of the peak identifying the target region, defined as the estimated false discovery rate (FDR) among the rows whose Q value is no larger than a chosen FDR. The NLQ value is defined as the -log_10_(Q value).

### Q-PCR

For ChIP-Seq target region validation, Q-PCR was performed using the Quantimix Easy SYG Kit (Biotools) (SYBR green I) in a iCycler iQ Multi-Color Real Time PCR Detection System (Bio-Rad). The enrichment of a promoter region in the ChIP sample was calculated as: enrichment = 2^[C_t_(sample)-C_t_(control)], where the input sample was used as the control. The efficiency of the PCR was calculated using the program LinRegPCR [49]. Primers used to amplify the *nrrA* and *all0770* promoters are indicated in Additional file 7: Table S5.

### Affinity purification of anti-NtcA

Anti-NtcA antibodies [50] were purified using the AminoLink Plus Immobilization Kit (Thermo Scientific) and purified NtcA protein. NtcA was purified as described [51].

### Electrophoretic mobility shift assays (EMSA)

The DNA fragments assayed were obtained by PCR using one of the primers labeled with T4 polynucleotide kinase (Roche) and [γ-^32^P] dATP. Additional file 7: Table S5 shows the primers used for each DNA fragment analyzed in Figure 3. 1–2 fmol of DNA was used in a final volume of 15 μl in binding buffer (10 mM Tris–HCl pH 8, 30 mM KCl, 10 mM MgCl_2_, 2 mM DTT, 5% glycerol). 0.04 mg/ml poly (dI-dC) and 0.04 mg/ml bobine serum albumine were used as non-specific competitor DNA and protein, respectively. The reaction mixtures with the corresponding DNA fragment were incubated with purified NtcA (100 nM and 400 nM) for 30 min at 30°C. The protein-DNA complexes were separated on native 8% polyacrylamide gels. Radioactive areas of the gels were visualized with a Cyclone storage phosphor systems (Packard).

## Availability of supporting data

The data sets supporting the results of this article, which can visualized with the Integrative Genome Viewer (Broad Institute), are available at GEO accession # GSE51865.

## Competing interests

The authors declare that they have no competing interests.

## Authors’ contributions

SP participated in the design of the experiments, performed the experiments, analyzed data, and drafted the manuscript. EF analyzed data and helped draft the manuscript. AH participated in the design of the experiments, analyzed data, and helped draft the manuscript. All authors read and approved the final manuscript.

## Supplementary Material

Additional file 1: Figure S1 Q-PCR verification of the immunoprecipitated material. Click here for file

Additional file 2: Table S1 Compendium of the binding regions found in the ChIP-Seq analysis. Click here for file

Additional file 3: Figure S2 Scheme of the ChIP-Seq results. Click here for file

Additional file 4: Table S2 Identification of previously described NtcA binding sites in *Anabaena* sp. PCC 7120 in ChIP-Seq target regions. Click here for file

Additional file 5: Table S3 Functional category of genes ascribed to target regions with NLQ>300. Click here for file

Additional file 6: Table S4 Comparison of the results of the ChIP-Seq analysis with the proposed NtcA-dependent genes by Su *et al.* and Mitschke *et al.* Click here for file

Additional file 7: Table S5 Primers used in this work. Click here for file
