# Supplementary material for: ChIP analysis unravels an exceptionally wide distribution of DNA binding sites for the NtcA transcription factor in a heterocyst-forming cyanobacterium
Source: BMC Genomics. 2014 Jan 13;15:22. doi: 10.1186/1471-2164-15-22 (PMC3898017; doi:10.1186/1471-2164-15-22)

Figure S1. Q-PCR verification of the immunoprecipitated material.
The enrichment of the promoter region of *all0770* as the negative control and of *nrrA* (*all4312)* as the positive control in the immunoprecipitated material was analyzed by Q-PCR. Primers used are indicated in the Table S5. Input, total DNA; ChIP, NtcA-immunoprecipitated DNA; Mock, DNA resulting from the whole ChIP protocol but with no anti-NtcA; Control, water. The mean from two independent ChIP experiments is shown.

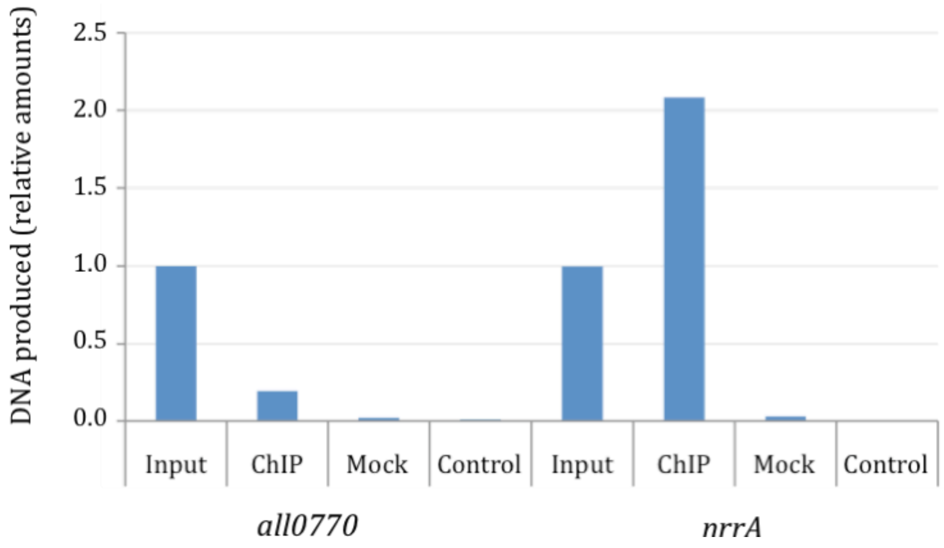

Supplement: Additional file 1: Figure S1 — Q-PCR verification of the immunoprecipitated material. [file 1471-2164-15-22-S1.pdf]
