# Supplementary material for: ChIP analysis unravels an exceptionally wide distribution of DNA binding sites for the NtcA transcription factor in a heterocyst-forming cyanobacterium
Source: BMC Genomics. 2014 Jan 13;15:22. doi: 10.1186/1471-2164-15-22 (PMC3898017; doi:10.1186/1471-2164-15-22)

Figure S2. Scheme of the ChIP-Seq results.

The results of the analysis of the massive sequencing data for the *Anabaena's* chromosome and plasmids are shown as visualized with the Integrative Genome Viewer program (Broad Institute, USA) (18). The raw coverage (CVG) at each target region is represented in the Y axis (the range of the CVG is indicated in parenthesis).

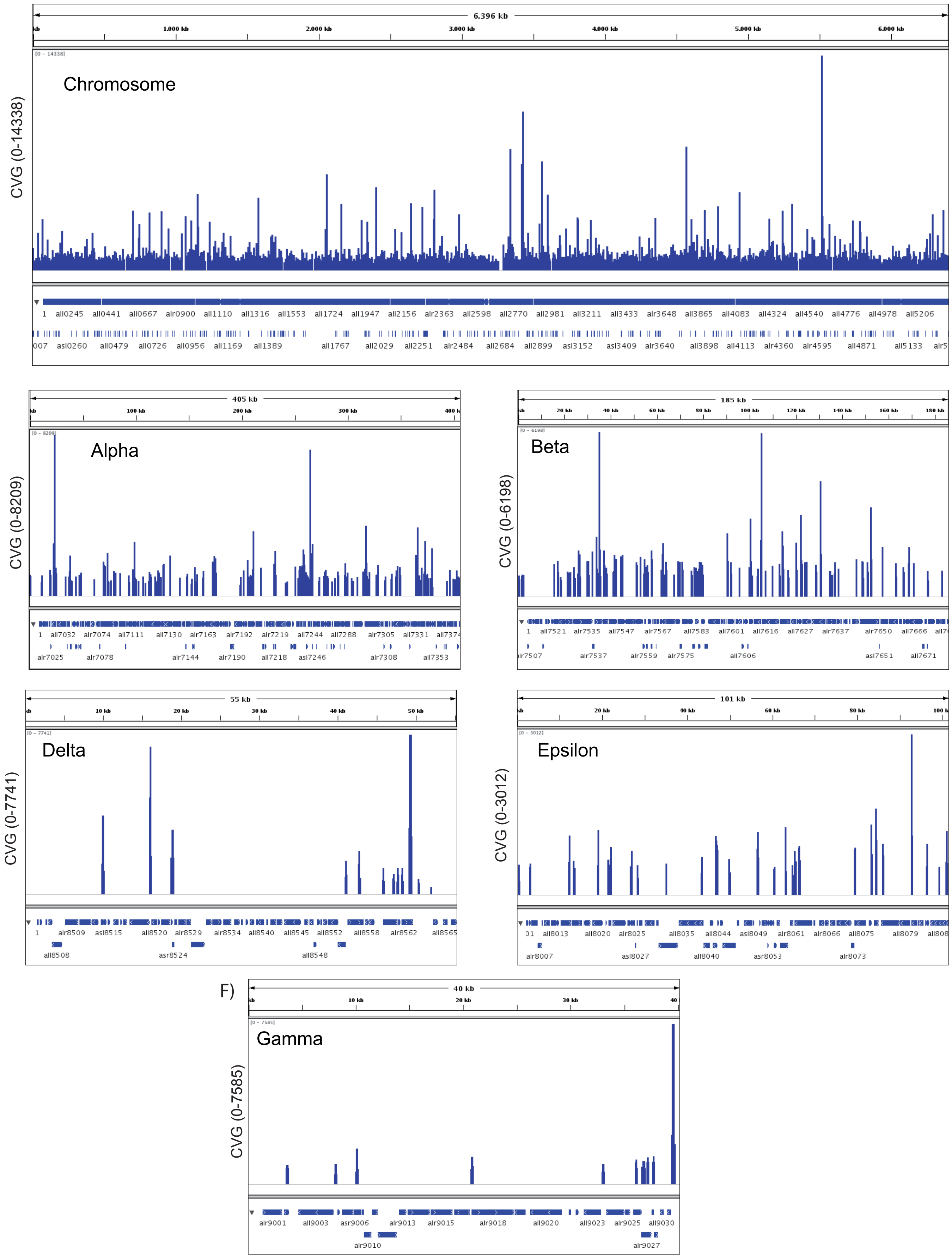

Supplement: Additional file 3: Figure S2 — Scheme of the ChIP-Seq results. [file 1471-2164-15-22-S3.pdf]
