# Supplementary material for: ChIP analysis unravels an exceptionally wide distribution of DNA binding sites for the NtcA transcription factor in a heterocyst-forming cyanobacterium
Source: BMC Genomics. 2014 Jan 13;15:22. doi: 10.1186/1471-2164-15-22 (PMC3898017; doi:10.1186/1471-2164-15-22)
Supplement: Additional file 5: Table S3 — Functional category of genes ascribed to target regions with NLQ>300. [file 1471-2164-15-22-S5.pdf]

Table S3. Functional categories of genes ascribed to target regions with NLQ>300

		Number of genes		
Category	Total	Upstream	Internal	Downstream
269 genes in total		134	129	6
Regulatory functions	33	10	23	
Hypothetical proteins	122	52	66	4
Heterocyst differentiation and function	13	9	4	1
Nitrogen Metabolism	12	11	1	
Amino acid biosynthesis	7	4	3	
Central Metabolism	8	4	4	
Transport and binding proteins	10	4	6	
Other categories	27	16	11	
Transcription+translation	12	9	3	
Others	25	15	9	1

The number of genes ascribed to the target regions with NLQ>300, grouped into different categories, is shown. There are 261 target regions with NLQ>300, which are ascribed to 269 genes. Positions of the target regions with regard to the genes to which they have been ascribed (upstream, internal and downstream) are indicated.
