# Supplementary material for: ChIP analysis unravels an exceptionally wide distribution of DNA binding sites for the NtcA transcription factor in a heterocyst-forming cyanobacterium
Source: BMC Genomics. 2014 Jan 13;15:22. doi: 10.1186/1471-2164-15-22 (PMC3898017; doi:10.1186/1471-2164-15-22)
Supplement: Additional file 7: Table S5 — Primers used in this work. [file 1471-2164-15-22-S7.pdf]

Table S5. Primers used in this work.

DNA tested	Primer	5'-3' Sequence
#204	all0438-1	ATATTTAAAACCCCCACAC
	all0438-2	CTGGTAAGGAAATTCGC
#259	all0602-16	CCAAATCTACCAAGCCATC
	all0602-17	GGAAGCAGCCAGAATTGTG
#364	all0926-1	CAATTGGTGGTGTCTGTTG
	all0926-2	GATTTCTGTCGTCGTCG
#602	all1517-1	TTCTTTGGGAGATTCGCC
	all1517-2	CATGCCAAGGAATTTCAGG
#892	alr2259-1	CTGGGCAGTTGAATCA
	alr2259-2	TTTGTCCTATAGGTTTAGTAGTG
#996	alr2482-1	CTTTTGGAATTTGGTACG
	alr2482-2	ATAAGTATCAATTAACAAAGGC
#1128	all2747-1	TTGATATTCTATACTATTTTGGC
	all2747-2	TTTCTACTTTTACCTCGTCC
#1135	all2756-1	CTAACGCTGCTTTAAGTTG
	all2756-2	GTTGTCACGCATAGAAGG
#1137	all2760-1	CAGATATAGTCCTCATGCTG
	all2760-2	GGAAATAAGGAATTGTGC
#1203	alr2921-1	GATTGATACCATGAATCTCC
	alr2921-2	TCACTTCACAATCCTTGC
#1570	all3880-1	AGCACTTGTACCTGCCAG
	all3880-2	TCGAAATTTTAGACATCCG
#1756	all4355-1	TCAGGATGATATTGCTGC
	all4355-2	AAAAACTGTCAAATGTGAAC
#605	alr1524-3	GAACTTTCAAAGAATAACTTATGC
	alr1524-4	GGTATTTAATGAATTGGGTTAG
#931	alr2328-22	CAAAGTTGACCCCTATGAG
	alr2328-23	TTGTTACTCCTTCTCTGCC
all2096	all2096-2	GTACATAAAAGCGAGGATAGG
	all2096-3	TAGTCGGCTATTTTATGTATACGAATTTG
A.h. psbA	C.K3-1	CAGAGCAGCCGATTGTCTGTTG
	C.K3-2	CTGATAAGTGAGCTATTCAC
nrrA internal	all4312-20	CCAAGGGGAGTTTATCGACC
	all4312-21	CCAACATTGCGGATAGTC
all0770 (Q-PCR)	all0770-3	GCTTAAAGTCTTTTCCCTATGTGCTTTC
	all0770-4	GCTTTTGGGAGGTTTTTTCTATCGT
nrrA promoter (Q-PCR)	nrrA-3	TTTGCCAACATAGGTTATAAAAAAACGTAG
	nrrA-4	AGACCCCTTAGCGTCGATGAAT
